# Improved 2D slice-interleaved flow-independent cardiac black blood imaging using Ferumoxytol

**DOI:** 10.1186/1532-429X-17-S1-Q21

**Published:** 2015-02-03

**Authors:** Junfei Lu, Paul J Finn, Peng Hu

**Affiliations:** 1 Bioengineering, University of California, Los Angeles, Los Angeles, CA, USA; 2 Radiology, University of California, Los Angeles, Los Angeles, CA, USA

## Background

Double inversion recovery (DIR) is the most commonly used black blood (BB) preparation method. However its single slice nature makes the acquisition inefficient and flow-dependence causes insufficient blood suppression when stagnant blood or in-plane blood flow is present. In this work, we propose to use ferumoxytol, an FDA approved iron oxide particle for treating iron deficiency anemia, as an MR contrast agent to achieve flow-independent BB imaging by taking advantage of its strong R2 relaxivity. As our technique eliminates the need for DIR preparation, we propose to achieve higher scan efficiency by interleaving the imaging slices.

## Methods

**Figure 1:**
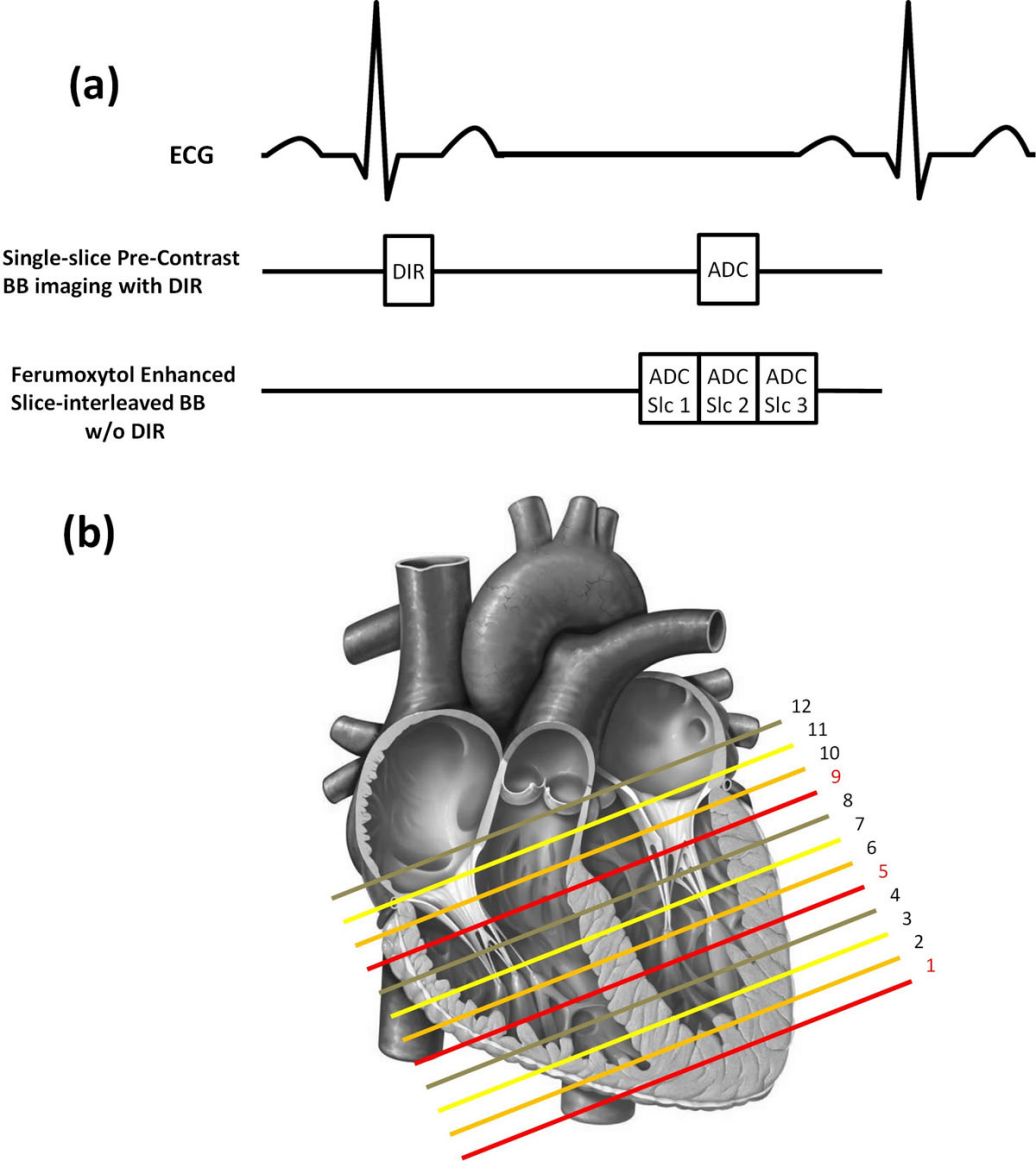
(a) Block diagrams for single-slice DIR TSE and slice-interleaved TSE without DIR. The removal of DIR pulses enable interleaved slice acquisition. The central ADC of the interleaved acquisition is located at the same cardiac phase as ADC in single slice scheme. (b) In traditional DIR BB imaging, 12 short axis slices are acquired sequentially one slice in each scan. While in the slice-interleaved mode, 3 slices with 300% slice distance are grouped into one interleaved acquisition. For example slice# 1,5,9 (marked in red) are acquired in one scan.

On seven healthy volunteers, we acquired 12 consecutive short axis (SA) slices covering the ventricles and 2 horizontal long axis (HLA) slices, both pre- and post- ferumoxytol injection. Conventional ECG gated single-slice breath-held DIR Turbo Spin Echo (TSE) sequence was used for pre-contrast acquisition, with TR=2 R-R interval, TE=37ms, echo spacing (ESP)=5.25 ms, echo train length (ETL)=15 and TI=600ms. Subsequently, ferumoxytol was injected (4 mg-Fe/kg) and the proposed BB imaging shown in Fig. 1 was performed. The elimination of DIR preparation enabled slice-interleaved scan where 12 SA slices were acquired in 4 breath-holds. TR, TE, ESP and ETL were kept the same for the post-contrast sequence. A TE of 37ms was found to be a good balance between suppressing blood signal and maintaining myocardium signal. SNR, CNR and sharpness of the septal wall were quantified and compared using the pre- and post-contrast BB techniques.

## Results

**Figure 2:**
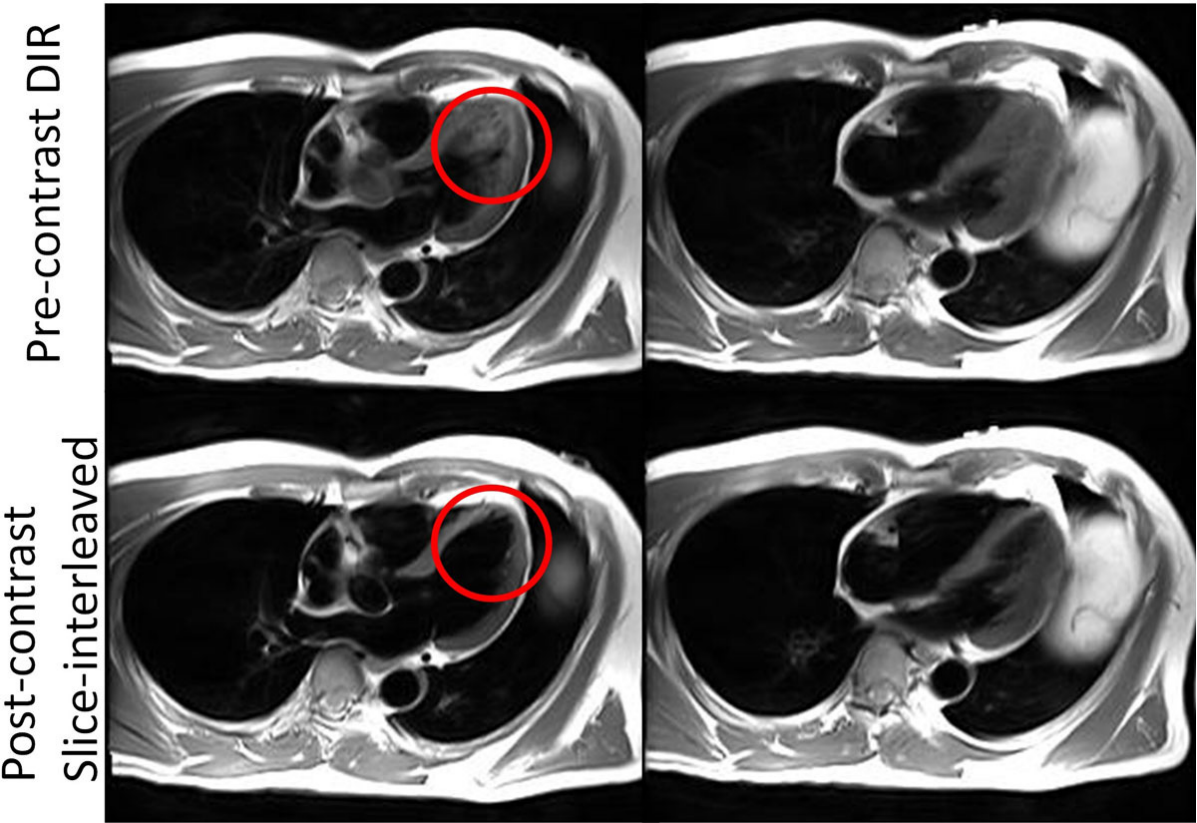
Two HLA views of the same volunteer with pre-contrast DIR images at top row and post-contrast slice-interleaved acquisition at bottom. The stagnant blood is evident in pre-contrast images near apex (Red circle) and mid-ventricular endocardium, whereas in post-contrast images, such residual signals are eliminated.

In mid-ventricle SA slices (slice number 7-12), both pre- and post- contrast images offered satisfactory dark blood contrast. For apical slices (slice number 1-6), post-contrast images showed significant improvement in all 7 volunteers by eliminating stagnant blood signal. In HLA views (shown in Fig. 2), the stagnant blood was evident in pre-contrast images near apex and mid-ventricle myocardium; whereas in post-contrast images, such residual blood signals were eliminated in all volunteers. Average myocardial SNR and myocardium-blood CNR increased after ferumoxytol injection, especially for apical segments (CNR: 6.2±8.13 vs. 22.6±6.4s, P<0.0001). The pre- and post-contrast BB imaging had similar sharpness values (P=0.877).

## Conclusions

Compared to conventional DIR BB imaging, the proposed ferumoxytol-enhanced slice-interleaved TSE technique provides improved blood signal suppression that does not depend on flow and is at least 3X faster than conventional DIR for the same anatomical coverage. It also provides higher SNR and CNR due to signal boost from ferumoxytol.

## Funding

N/A.

